# Pharmacological regulation of airway ciliary motility: endogenous mediators, pharmaceutical agents, and natural compounds with a focus on ginsenoside Rd

**DOI:** 10.1007/s11418-026-02039-0

**Published:** 2026-04-30

**Authors:** Kouta Noriyama, Eishi Ashihara

**Affiliations:** Laboratory of Clinical and Translational Physiology, Kyoto Pharmaceutical University, 5 Nakauchi-cho, Misasagi, Yamashina-ku, Kyoto, 607–8414 Japan

**Keywords:** Ciliary motility, Mucociliary clearance, Natural compound: Ginsenoside Rd

## Abstract

Mucociliary clearance (MCC) is a central component of airway innate defense, and its impairment contributes to respiratory morbidity in chronic obstructive pulmonary disease, asthma, primary ciliary dyskinesia, and chemotherapy-induced ciliary dysfunction. Reduced ciliary motility increases susceptibility to infection, making pharmacological enhancement of airway cilia an attractive yet underexplored strategy. Ciliary activity is regulated by intracellular signaling pathways including cAMP-, Ca^2+^-, nitric oxide-, and Cl⁻- signaling, which converge on axonemal dynein to control ciliary beat frequency, bend angle, and bend distance. Although numerous endogenous mediators, pharmaceutical agents, and natural compounds stimulate ciliary motility, these findings remain fragmented and lack systematic integration. This review classifies airway ciliary activators by signaling mechanism and discusses their translational relevance in diseases characterized by impaired MCC. Particular emphasis is placed on ginsenoside Rd as a representative natural compound that enhances ciliary motility through activation of estrogen receptor β and potentiation of the P2 X 7 receptor and ameliorates chemotherapy-induced ciliary motility disturbance. Its preferential lung accumulation supports translational potential. By integrating intracellular signaling mechanisms with translational perspectives, this review highlights pharmacological activation of airway cilia, including strategies derived from natural compounds and herbal medicines, as a meaningful and underexplored approach to enhancing MCC, and outlines key knowledge gaps and future research directions.

**Graphical abstract:**

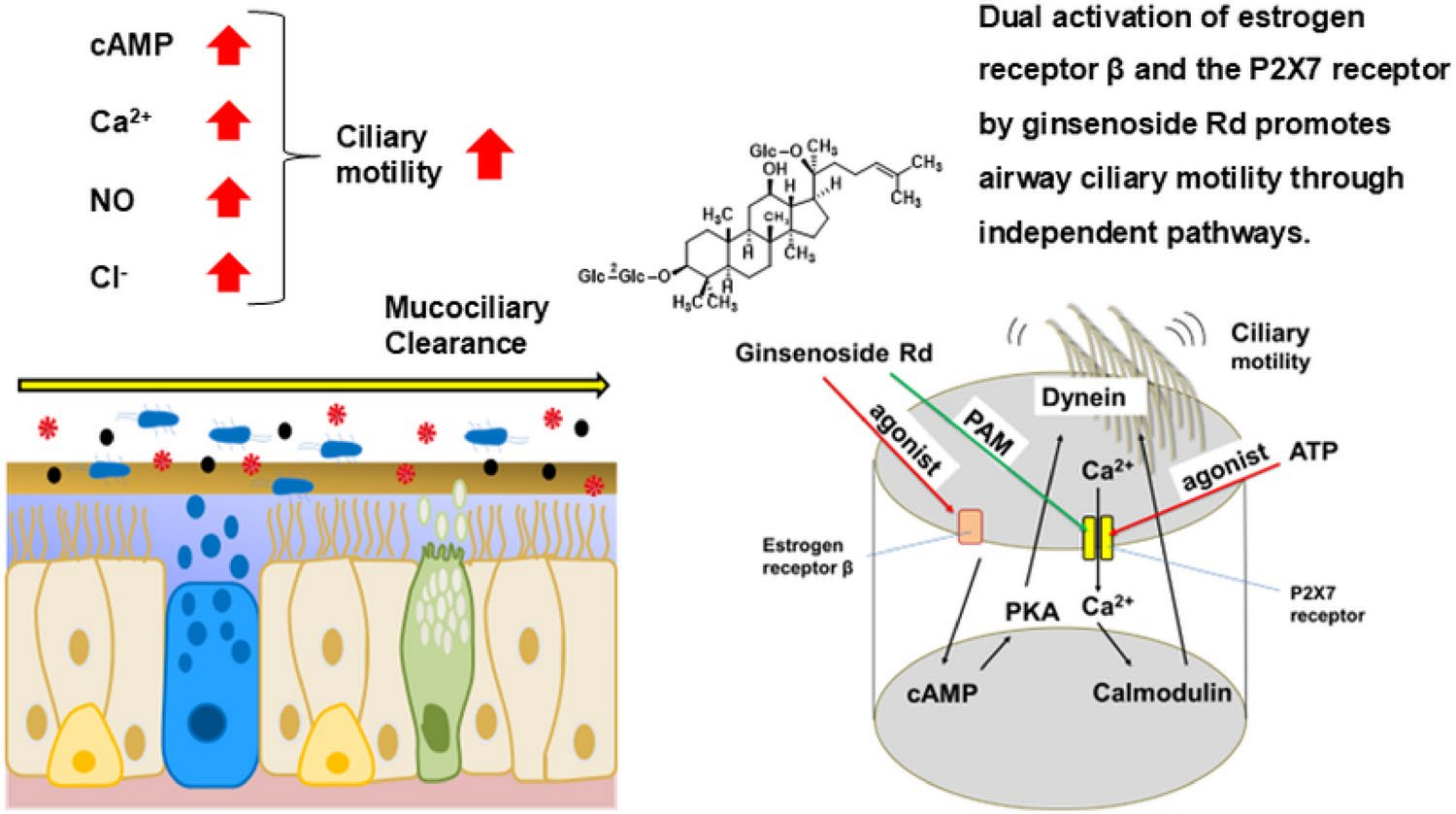

## Introduction

The air we constantly inhale contains a variety of foreign substances, including bacteria, fungi, viruses, and fine particulate matter 2.5 (PM2.5). Reported bacterial concentrations range from 8.71 × 10³ to 2.14 × 10⁷ cells/m³ in air [1]. These bacteria can reach the airway surfaces, causing cellular damage and triggering infections. In 2019, lower respiratory tract infections affected approximately 480 million people and resulted in 2.4 million deaths worldwide [2]. Consequently, respiratory infections remain a significant global health concern.

To protect against these inhaled threats, the human airway is equipped with mucociliary clearance (MCC). MCC, composed of mucus secretion by goblet cells and the coordinated beating of ciliated cells, efficiently transports foreign substances out of the respiratory tract. MCC is an important innate immune mechanism that prevents the entry of pathogenic materials into the respiratory tract [3]. The function of MCC is commonly assessed by measuring ciliary motility and mucus secretion. Ciliary motility is clinically critical because it is directly involved in the removal of foreign substances. Ciliary motility is commonly quantified using parameters such as ciliary beat frequency (CBF), ciliary bend angle (CBA), and ciliary bend distance (CBD). CBF represents the number of ciliary beats per unit time, CBA indicates the bending angle of the cilium, and CBD reflects the displacement of the distal tip of the cilium [4, 5]. Among these, CBF is the most widely used and informative indicator of MCC activity.

Impaired ciliary motility is associated with an increased susceptibility to airway infections in various respiratory and clinical conditions [6]. Accordingly, pharmacological strategies that enhance ciliary motility are considered a promising approach to strengthen airway defense. Although an increasing number of compounds have been reported to stimulate ciliary beating, the underlying regulatory mechanisms remain poorly integrated. Several reviews have addressed the physiology of ciliary beating or the molecular regulation of mucociliary clearance. However, few have systematically focused on endogenous mediators, pharmacological agents, and natural compounds that enhance ciliary activity from a mechanistic perspective.

In this review, we summarize the regulatory mechanisms of airway ciliary motility, focusing on endogenous mediators, pharmacological agents, and natural compounds that enhance ciliary function, with particular emphasis on the mechanisms of action of ginsenoside Rd (Rd). In addition, we provide a comprehensive overview of the structure and function of airway ciliated cells and their relevance to respiratory diseases and therapeutic strategies, highlighting key knowledge gaps and future research directions.

## Cellular composition and physiological roles of the mucociliary clearance system

MCC is maintained through the coordinated activity of various cell types in the airway epithelium [3]. MCC serves as a crucial immune defense mechanism against inhaled pathogens and foreign particles. This section provides an overview of the cellular components of MCC, the molecular mechanisms that regulate ciliary motility, and the consequences of MCC impairment for respiratory health.

### Cellular components of MCC

**Fig. 1:**
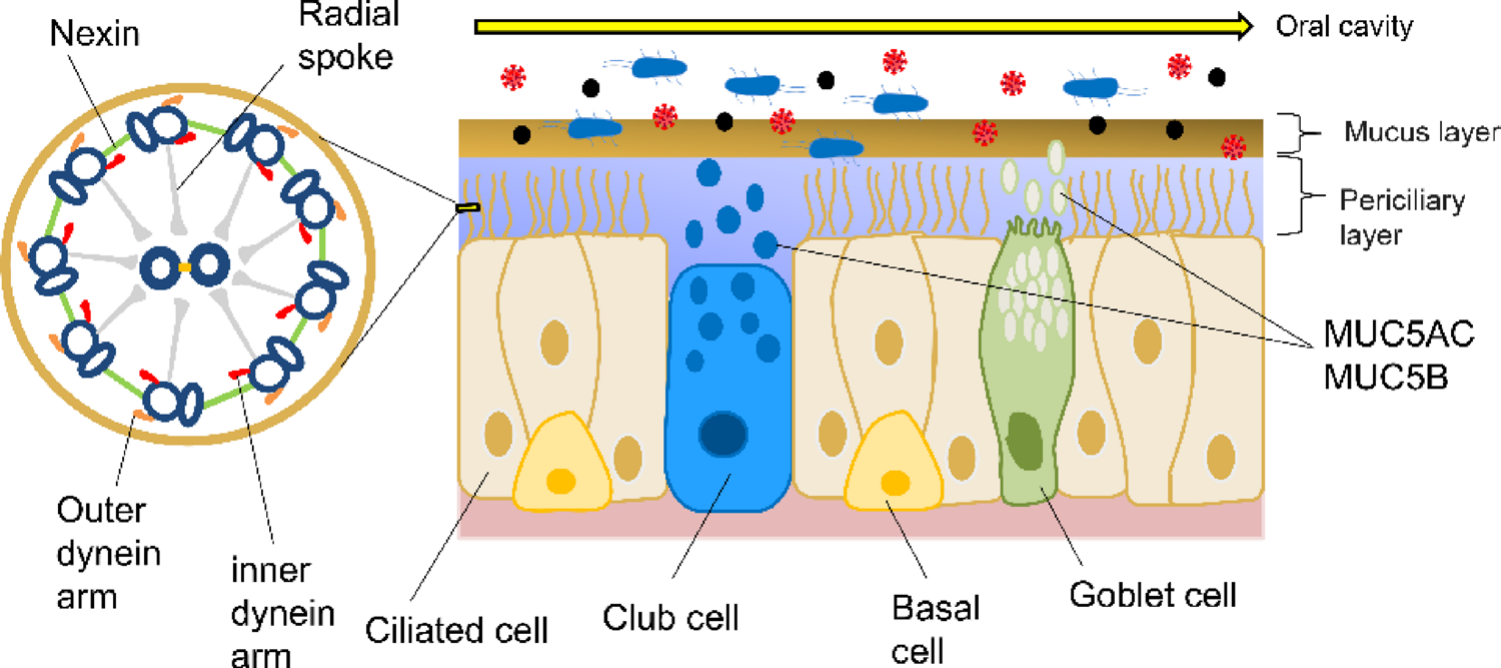
Structure and components of the airway mucociliary clearance (MCC) system. MCC is regulated by ciliated and goblet cells within the airway epithelium. The apical surface is covered by a two-layered system consisting of the watery periciliary layer and the viscous mucus layer. Cilia (left) display a “9 + 2” arrangement, composed of nine doublets and a central pair. Dynein motor proteins (inner and outer dynein arms) coordinate ciliary beating, which drives the mucus layer and entrapped foreign particles toward the oral cavity. Goblet cells and club cells secrete mucins, including MUC5AC and MUC5B, forming the adhesive mucus layer. Basal cells are epithelial stem cells that replenish these specialized cell types

This section describes the major cell types of the airway epithelium and their distinct contributions to MCC. MCC is composed of multiple specialized cell types that perform distinct roles. The main cellular constituents are basal cells, club cells, goblet cells, and ciliated cells [3] (Fig. 1). Basal cells function as epithelial stem cells and are regulated by Notch signaling to differentiate into ciliated or goblet cells [7]. Club cells produce secretory proteins such as SCGB1B1 and mucins, including MUC5B [8]. Club cells also serve as progenitors that can differentiate into ciliated or goblet cells [7]. Goblet cells are the main source of mucins, secreting MUC5AC and MUC5B [9]. These mucins contribute to pathogen trapping through cross-linking and glycosylation of their side chains [10]. Ciliated cells have hundreds of motile cilia on their apical surface. These cilia generate the mechanical force necessary to transport mucus toward the oral cavity. Ciliary activity is assessed using parameters such as CBF, CBA, and CBD [5].

### Molecular and signaling mechanisms regulating ciliary motility

This section summarizes the key structural features and signaling pathways that regulate ciliary beating. The structure and molecular mechanisms of motile cilia have been previously described [11]. Therefore, they are summarized only briefly here. Motile cilia possess a conserved axonemal structure, forming the characteristic “9 + 2” arrangement [11] (Fig. 1).

**Fig. 2:**
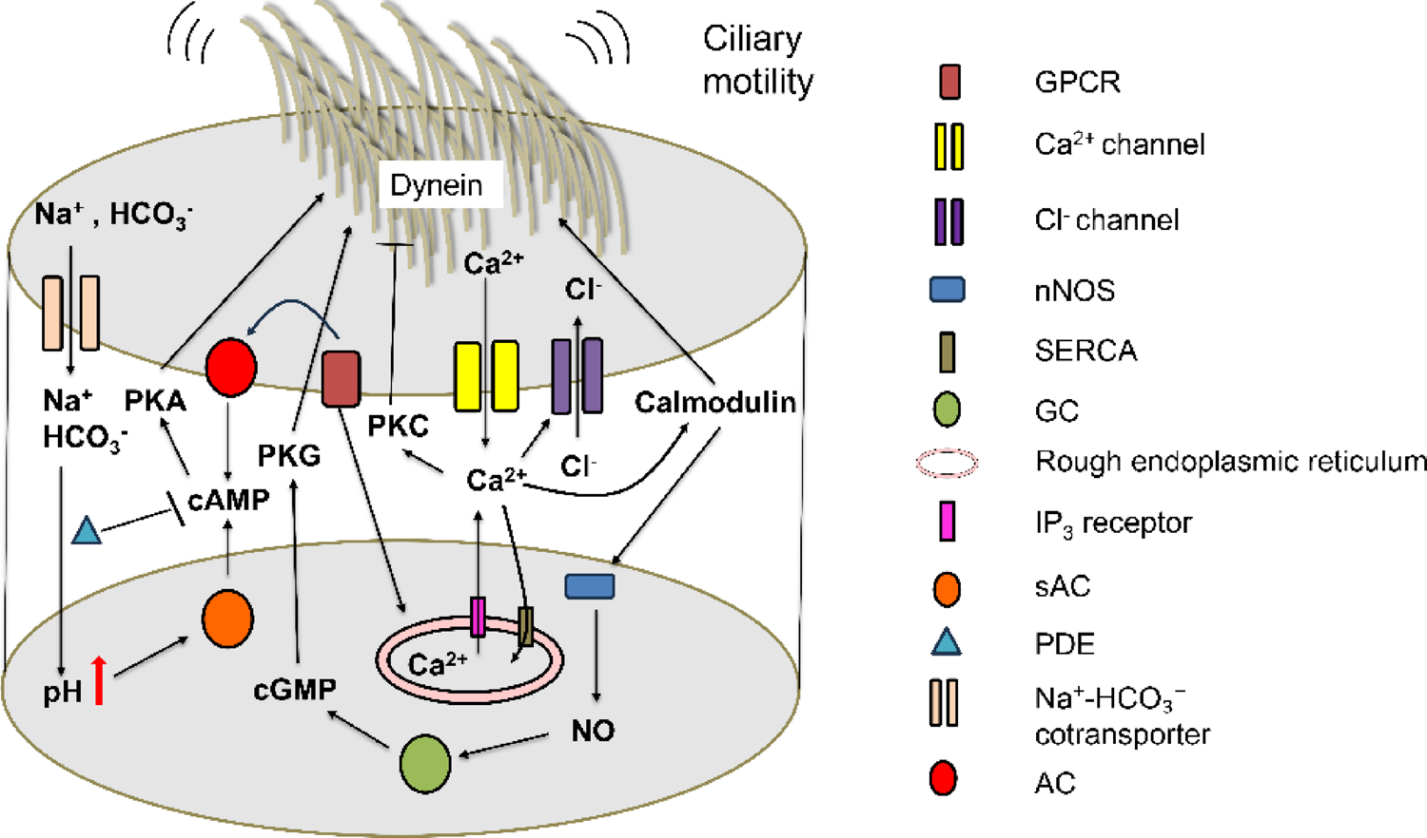
Molecular and signaling mechanisms regulating airway ciliary motility. Major signaling pathways involved in regulating ciliary motility. Ciliary motility is regulated by multiple signaling cascades, with the central mechanism involving the phosphorylation and activation of the motor protein dynein. (1) cAMP/protein kinase A (PKA) Pathway: G protein-coupled receptors (GPCR; e.g., β2-adrenergic receptor, estrogen receptor, prostaglandin I receptor, P2Y receptor, muscarinic receptor) increase intracellular cAMP levels via the activation of adenylyl cyclase (AC), thereby stimulating PKA. Soluble adenylyl cyclase (sAC), which is pH-sensitive, also contributes to cAMP production. cAMP is degraded by phosphodiesterase (PDE). (2) Ca^2+^ dependent Pathways: elevation of intracellular Ca^2+^ ([Ca^2+^]_i_) level is induced by extracellular Ca^2+^ influx through ionotropic channels (e.g., P2X receptor, nicotinic receptor, taste family type 2 receptors, voltage-dependent Ca^2+^ channel, transient receptor potential vanilloid 4) or the release from the rough endoplasmic reticulum through the IP_3_ receptor. Increased [Ca^2+^]_i_ activates calmodulin that regulates dynein activity. [Ca^2+^]_i_ is maintained by SERCA (Sarco/endoplasmic reticulum Ca^2+^-ATPase). Ca^2+^ can also activate protein kinase C (PKC), which is generally reported to suppress ciliary motility. (3) NO/protein kinase G (PKG) Pathway: Ca^2+^-calmodulin activates neuronal nitric oxide synthase (nNOS), producing NO. NO activates increase intracellular cGMP levels via the activation of guanylyl cyclase (GC), thereby stimulating PKG. (4) Cl^–^ Regulation: [Ca^2+^]_i_ influences Cl^–^ channels (e.g., cystic fibrosis transmembrane conductance regulator, anoctamin-1). A reduction in intracellular Cl^–^ levels is known to induce an increase in [Ca^2+^]_i_ level

The inner dynein arms, outer dynein arms, nexin, and radial spokes are key structural components of motile cilia [11] (Fig. 1). These axonemal structures function in a highly coordinated manner to generate effective ciliary beating. Detailed descriptions of their molecular composition and mechanical roles have been provided in previous reports and are not repeated here [11, 12]. Dynein generates ciliary beating through ATP hydrolysis, but its upstream regulatory mechanisms remain incompletely understood. Importantly, phosphorylation of dynein arms plays a critical role in modulating the speed and force of ciliary beating. Key signaling pathways implicated in dynein phosphorylation include cAMP/protein kinase A (PKA), Ca^2+^/calmodulin, nitric oxide (NO)/protein kinase G (PKG), pH-sensitive soluble adenylyl cyclase (sAC), and Cl⁻ channels (Fig. 2). An increase in intracellular cAMP concentration ([cAMP]_i_) activates PKA, thereby enhancing ciliary motility [13]. Elevations in intracellular Ca^2+^ concentration ([Ca^2+^]_i_) are suggested to regulate dynein activity through calmodulin-dependent kinases [14], while also exerting inhibitory effects on CBF via protein kinase C (PKC) activation [15, 16]. Chronic Ca^2+^ stimulation, such as aging or smoking, is thought to induce the activation of PKC rather than calmodulin [15, 17]. Ca^2+^–calmodulin signaling further stimulates NO production through activation of neuronal nitric oxide synthase [18], leading to PKG activation [19, 20]. [Ca^2+^]_i_ may also enhance ciliary motility by activating Cl⁻ channels, triggering additional Ca^2+^ influx [21, 22]. Although the detailed mechanisms are not fully understood, these pathways likely cooperate to regulate CBF and CBA.

### Impairment of MCC and increased infection risk

Here, we discuss how impaired ciliary motility compromises MCC and increases susceptibility to respiratory infections. Impaired ciliary motility directly reduces MCC and markedly increases the risk of respiratory infections. In primary ciliary dyskinesia (PCD), ciliary motility is impaired [23]. As a result, PCD leads to an elevated risk of respiratory infections due to compromised MCC [24]. In bronchial asthma and chronic obstructive pulmonary disease (COPD), chronic airway inflammation causes airway remodeling, mucus hypersecretion, and a reduction in ciliary motility [25, 26]. Consequently, patients with asthma have been reported to be up to 1.3-fold more susceptible to upper respiratory tract infections and 2.9-fold more susceptible to lower respiratory tract infections, and patients with COPD exhibit an approximately 4.8-fold higher risk of pneumonia compared with healthy individuals [27, 28]. In recent years, cancer therapies have also been reported to increase the risk of respiratory infections [29]. Therefore, enhancing ciliary motility is a key aspect of airway defense. Strategies that enhance ciliary motility may help prevent respiratory infections, particularly in patients with impaired MCC.

## Activators of ciliary motility

This section reviews endogenous mediators, pharmaceutical agents, and natural compounds that enhance ciliary activity, with a focus on their signaling mechanisms. The regulation of ciliary motility involves not only endogenous mediators but also clinically used pharmaceutical agents and diverse natural compounds. These substances are known to exert their effects through signaling pathways such as Ca^2+^, cAMP, NO, and Cl⁻. In this section, representative examples are presented, focusing on the signaling mechanisms by which each compound enhances ciliary activity.

### Endogenous mediators as activators of ciliary motility

This section describes how endogenous mediators, including hormones and autacoids, regulate ciliary motility via the rapid activation of G protein-coupled receptors (GPCR) or ionotropic receptors on ciliated cells (Table 1).

**Table 1:** Endogenous mediators that enhance ciliary motility

Compound	Cell Type	Change of CBF regulator	Effect	References
ATP	Rat tracheal (ex vivo), Mouse tracheal and bronchial (ex vivo)	[Ca^2+^]_i_ ↑	CBF ↑ CBA ↑	[30, 88, 89]
Acetylcholine	Rat tracheal (ex vivo), Mouse airway (ex vivo)	[Ca^2+^]_i_ ↑	CBF ↑ CBD ↑	[32, 33]
E2	Mouse airway (ex vivo)	[cAMP]_i_ ↑	CBF ↑	[36, 75]
Prostaglandin E2	Human nasal (in vitro)	[cAMP]_i_ ↑	CBF ↑	[38, 39]

#### Ca^2+^-dependent signaling by endogenous mediators

Physiological stimuli selectively modulate intracellular signaling to control MCC. ATP acts on ionotropic P2X receptors and G protein–coupled P2Y receptors in ciliated cells [30]. These receptors induce increases in CBF and CBA through extracellular Ca^2+^ influx and the release of Ca^2+^ from rough endoplasmic reticulum stores into cytosol.

Acetylcholine (ACh) also activates ciliary motility via Ca^2+^-dependent signaling. ACh increases [Ca^2+^]_i_ through Ca^2+^ release from intracellular stores and voltage-dependent Ca^2+^ channels via muscarinic (M1, M3) and nicotinic (α7) ACh receptors, resulting in increased CBF and CBD [31, 32]. Furthermore, ACh also promotes ATP release under hypotonic conditions, resulting in a further increase in CBF [33].

#### cAMP-dependent signaling by endogenous mediators

Estrogen receptors (ER) are localized at both the plasma membrane and the cytosol, mediating non-genomic and genomic actions, respectively [34, 35]. In ciliated cells, 17β-Estradiol (E2) enhances ciliary activity via membrane ERα, ERβ [36, 37]. In particular, activation of membrane ERβ has been reported to increase CBF through elevation of [cAMP]_i_ and subsequent activation of PKA [37]. Prostaglandin E2 similarly increases CBF via [cAMP]_i_- dependent signaling, whereas thromboxane A2 does not significantly affect CBF [38, 39]. These findings suggest selective prostanoid responsiveness in ciliated cells.

#### NO-dependent signaling by endogenous mediators

Evidence that endogenous mediators stimulate ciliary motility via an NO-dependent pathway remains limited. Ciliated cells can produce NO, which activates downstream PKG in an autocrine manner [40]. This pathway has also been suggested to be engaged in response to stimulation by foreign substances [41].

### Pharmaceutical agents as activators of ciliary motility

This section describes how clinically used drugs enhance ciliary motility through well-defined molecular pathways. Synthetic drugs often have well-defined targets, whereas chinese herbal medicines affect multiple signaling pathways because they contain diverse bioactive components. Both types can effectively enhance ciliary motility (Table 2).

**Table 2:** Pharmaceutical agents that enhance ciliary motility

Compound	Cell Type	Change of CBF regulator	Effect	References
Ambroxol	Mouse airway (ex vivo)	[Ca^2+^]_i_ ↑ [Cl^−^]_i_ ↓ pH_i_ ↑	CBF ↑ CBA ↑ CBD ↑	[22, 42]
Carbocisteine	Mouse airway (ex vivo), Human nasal (in vitro)	[Cl^−^]_i_ ↓ pH_i_ ↑	CBF ↑ CBA ↑ CBD ↑	[43, 44]
β2-adrenergic agonist	Human bronchial (in vitro), Rat airway (ex vivo)	[cAMP]_i_ ↑	CBF ↑	[47, 90]
Roxithromycin, Erythromycin	Rabbit airway (ex vivo)	[cAMP]_i_ ↑	CBF ↑	[49]
Iloprost	Human nasal (in vitro)	[cAMP]_i_ ↑	CBF ↑	[39]
Hochu-ekki-to	Mouse airway (ex vivo)	[Ca^2+^]_i_ ↑	CBF ↑ CBA ↑	[45]
Seihai-to	Mouse airway (ex vivo)	[Ca^2+^]_i_ ↑ [cAMP]_i_ ↑	CBF ↑ CBA ↑	[46]

#### Ca^2+^and Cl–dependent signaling by pharmaceutical agents

It has been reported that ambroxol increases CBF, CBA, and CBD by raising [Ca^2+^]_i_ level, decreasing intracellular Cl⁻ ([Cl⁻]_i_) level, and elevating intracellular pH (pH_i_) [22, 42]. These effects are considered to be initiated by extracellular Ca^2+^ influx via voltage-dependent Ca^2+^ channel CaV1.2, subsequently stimulating the Na⁺-HCO₃⁻ cotransporter (NBC) and anoctamin-1, leading to an increase in pH_i_ and a decrease in [Cl⁻]_i_ [22, 42]. Carbocisteine exhibits similar effects on [Cl⁻]_i_ and pH_i_, and its mechanism has been suggested to involve cystic fibrosis transmembrane conductance regulator (CFTR) and NBC, although it remains incompletely characterized [43, 44]. Hochu-ekki-to enhances CBF and CBA via an increase in [Ca^2+^]_i_ mediated by transient receptor potential vanilloid 4 activation [45]. Seihai-to has also been reported to increase CBF and CBA [46]. Although an elevation in [Ca^2+^]_i_ has been observed, the molecular mechanism underlying this increase has yet to be elucidated.

#### cAMP-dependent signaling by pharmaceutical agents

β2-adrenergic agonists such as salbutamol enhance ciliary motility via elevation of [cAMP]_i_ [47]. β2-adrenergic receptors are Gs-coupled GPCR, and the resulting activation of downstream PKA leads to an increase in CBF [48]. Roxithromycin and erythromycin, both macrolide antibiotics, and iloprost, a prostacyclin analog, also enhance CBF through [cAMP]_i_-dependent pathways [39, 49]. The mechanism by which roxithromycin and erythromycin increase [cAMP]_i_ remains unclear. Iloprost is thought to act via the IP receptor, a Gs-coupled GPCR. Seihai-to has also been reported to raise [cAMP]_i,_ suggesting that inhibition of phosphodiesterase (PDE) 1 is involved in its effects [46].

### Natural compounds as activators of ciliary motility

Here, we discuss representative natural compounds that increase ciliary motility and their underlying molecular mechanisms. Natural compounds are recognized as potent candidates for the activation of ciliary motility. Similar to endogenous mediators and pharmaceutical agents, many natural compounds exert their effects through signaling pathways involving Ca^2+^, cAMP, NO, and Cl⁻ (Table 3).

**Table 3:** Natural compounds that enhance ciliary motility

Compound	Cell Type	Change of regulator	Effect	References
Forskolin	Human airway (in vitro)	[cAMP]_i_ ↑	CBF ↑	[53, 91]
Genistein	Human nasal (in vitro)	[Cl^−^]_i_ ↓	CBF ↑	[57]
Daidzein	Human nasal (in vitro)	[Cl^−^]_i_ ↓	CBD ↑	[58]
Quinine	Human nasal (in vitro)	NO ↑	CBF ↑	[51]
Apigenin, chrysin	Human nasal (in vitro)	[Ca^2+^]_i_ ↑ NO ↑	CBF ↑	[50]
*Drosera rotundifolia* L. extract	Mouse airway (ex vivo)	[cAMP]_i_ ↑	CBF ↑	[54]
Quercetin, 2″-O-Galloylhyperoside	Mouse airway (ex vivo)	[cAMP]_i_ ↑	CBF ↑	[54]
Thyme extract	Human airway (COPD patients; in vivo)	[cAMP]_i_ ↑ [Ca^2+^]_i_ ↑	CBF ↑	[55]
Naringenin	Rat tracheal (ex vivo)	[cAMP]_i_ ↑	CBF ↑	[56]
Cynaropicrin	Human airway (Sarcoidosis or lung cancer patients; in vivo)	[Ca^2+^]_i_ ↑	CBF ↑	[52]
Red ginseng aqueous extract	Mouse nasal (ex vivo)	[Cl^−^]_i_ ↓	CBF ↑	[59]
Ginsenoside Rd	Mouse airway (ex vivo)	[cAMP]_i_ ↑ [Ca^2+^]_i_ ↑ (as positive allosteric modulator of P2X7)	CBF ↑	[70]

#### Ca^2+^and NO-dependent signaling by natural compounds

Many natural compounds activate ciliary motility through an elevation of [Ca^2+^]_i_, frequently mediated by the bitter taste receptor, or taste family type 2 receptors (T2R) 14. Flavonoids such as apigenin, derived from bee propolis, and chrysin, derived from *Passiflora* flowers, have been reported to enhance CBF via increases in [Ca^2+^]_i_ and NO production by stimulating T2R14 [50]. It has also been suggested that quinine stimulates T2R14, resulting in increased NO production and CBF [51]. These reports indicated that T2R14 not only directly mediates Ca^2+^ signaling but also engages the NO-PKG pathway to enhance CBF. On the other hand, cynaropicrin increases CBF by elevating [Ca^2+^]_i_ through a unique mechanism independent of T2R. Cynaropicrin has been reported to inhibit the sarco/endoplasmic reticulum Ca^2+^-ATPase, thereby inducing store-operated calcium entry, which subsequently leads to an increase in [Ca^2+^]_i_ and CBF [52].

#### cAMP-dependent signaling by natural compounds

Forskolin is a typical example of a natural compound that enhances ciliary motility. Forskolin increases [cAMP]_i_ by directly activating membrane adenylyl cyclase, and it has been reported to enhance CBF via PKA activation [53]. Extracts of *Drosera rotundifolia* L. and flavonoids such as quercetin, hyperoside, and 2″-O-galloylhyperoside, isolated from *Drosera rotundifolia* L., have been reported to enhance CBF via elevation of [cAMP]_i_ [54]. These effects are suggested to be mediated by the inhibition of PDE 1 A and 4D. Additionally, extracts of thyme and the flavonoid naringenin, contained in citrus fruits and grapes, have also been reported to enhance ciliary activity by increasing [cAMP]_i_ [55, 56]. However, the precise mechanisms underlying these effects have not yet been fully elucidated.

#### Cl–dependent signaling by natural compounds

Decreased [Cl⁻]_i_ indirectly enhances ciliary motility. Isoflavones such as daidzein increase CBD, and genistein increases CBF, with genistein potentially acting via CFTR [57, 58]. Daidzein does not significantly alter [cAMP]_i_ or [Ca^2+^]_i_, suggesting a main effect through CFTR-mediated [Cl⁻]_i_ reduction. Red ginseng aqueous extract (RGAE) also reduces [Cl⁻]_i_ and increases CBF by stimulating anoctamin-1 independently of CFTR. This finding may indicate the potential usefulness of RGAE in cystic fibrosis [59, 60].

Among various natural compounds reported to enhance ciliary motility, ginsenosides derived from ginseng have attracted attention because of their unique steroid-like triterpene structures, well-characterized pharmacokinetics, and reported respiratory benefits, such as anti-inflammatory, antioxidant, and antifibrotic effects [61–63]. Therefore, the following section focuses on the pharmacological actions and potential mechanisms of ginsenosides as ciliary activators.

## Ginsenosides as potential ciliary activators

This section focuses on ginsenosides, particularly Rd, highlighting their chemical properties, mechanisms of action, and potential roles as activators of airway ciliary motility.

### Structural classification and pharmacokinetic characteristics of ginsenosides

**Fig. 3:**
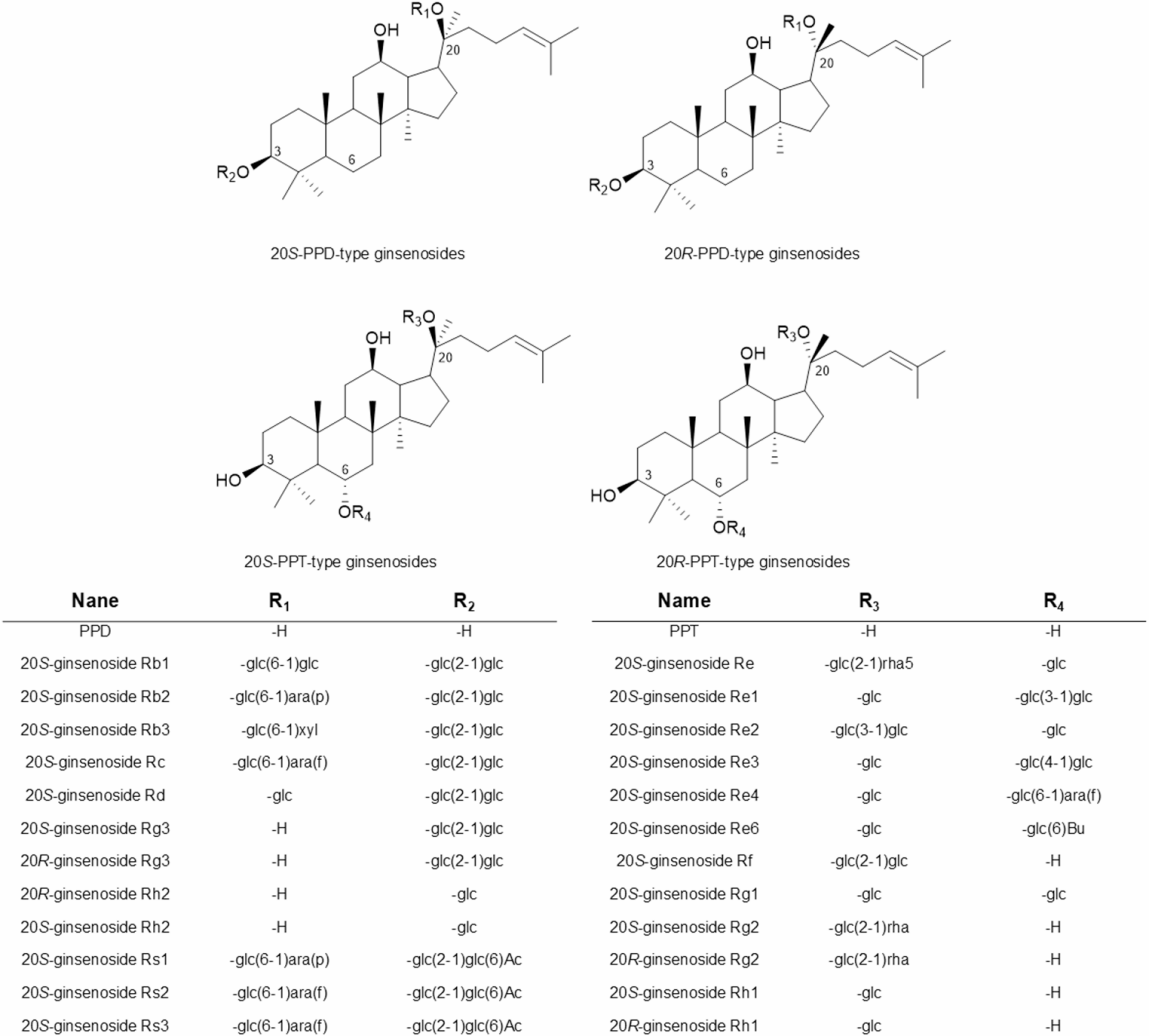
Chemical structures and classification of major ginsenosides. The core skeletons of protopanaxadiol (PPD) and protopanaxatriol (PPT) ginsenosides are shown. Variable substituents at each position are indicated as “R”. The table summarizes representative ginsenosides and their corresponding substituents. Abbreviations: ara(f): α-L-arabinofuranosyl; ara(p): α-L-arabinopyranosyl; glc: β-D-glucopyranosyl; xyl: β-D-xylopyranosyl; rha: α-L-rhamnopyranosyl; Ac: acetyl; Bu: trans-but-2-enoyl. This figure was created by the authors and adapted from previous studies on ginsenoside structures [64]

This subsection describes the chemical classification of ginsenosides, highlighting structural differences that influence their pharmacokinetics and biological activities. Ginsenosides are triterpene saponins with steroid-like backbones. They are classified into protopanaxadiol (PPD)-type and protopanaxatriol (PPT)-type. Representative ginsenosides and their core structures are summarized in Fig. 3, adapted from [64]. PPD-type of ginsenosides possess a hydrogen atom at the C-3, C-20 positions, with sugar chains attached. In contrast, PPT-type ginsenosides typically feature a hydroxyl group at C-6, C-12, and C-20 positions, with sugar chains bound to C-6 and C-20 [64]. These structural differences lead to variations in pharmacokinetics, receptor binding properties, and pharmacological activities. The composition of ginsenosides also varies depending on the plant part. In hydroponically cultivated *Panax ginseng* C. A. Meyer, the roots contain the highest levels of both PPD and PPT ginsenosides, whereas leaves and stems contain moderate amounts [65]. The major ginsenosides ginsenoside Rb1, Rb2, Rc, and Rd belong to the PPD type. Ginsenoside Rg1 and Re are classified as PPT-type compounds. PPD-type ginsenosides tend to be relatively stable in the body, whereas PPT-type ginsenosides are more rapidly eliminated [64].

### Distribution of ginsenosides and implications for MCC modulation

This subsection summarizes the absorption and tissue distribution of major ginsenosides, emphasizing pulmonary delivery and lung accumulation as key factors for their potential to modulate MCC. Understanding the absorption and tissue distribution of major ginsenosides is crucial for discussing their pharmacological effects. When assessing their potential as MCC modulators, pulmonary delivery represents a critical consideration. In vivo rodent studies have shown that both PPD and PPT ginsenosides reach relatively high concentrations in the lungs [66]. Notably, the PPD-type Rd has been reported to accumulate in lung tissue following both intravenous and oral administration in mice and rats, suggesting its potential to modulate MCC in vivo [67, 68].

### Molecular mechanisms of enhancement of ciliary motility by Rd

**Fig. 4:**
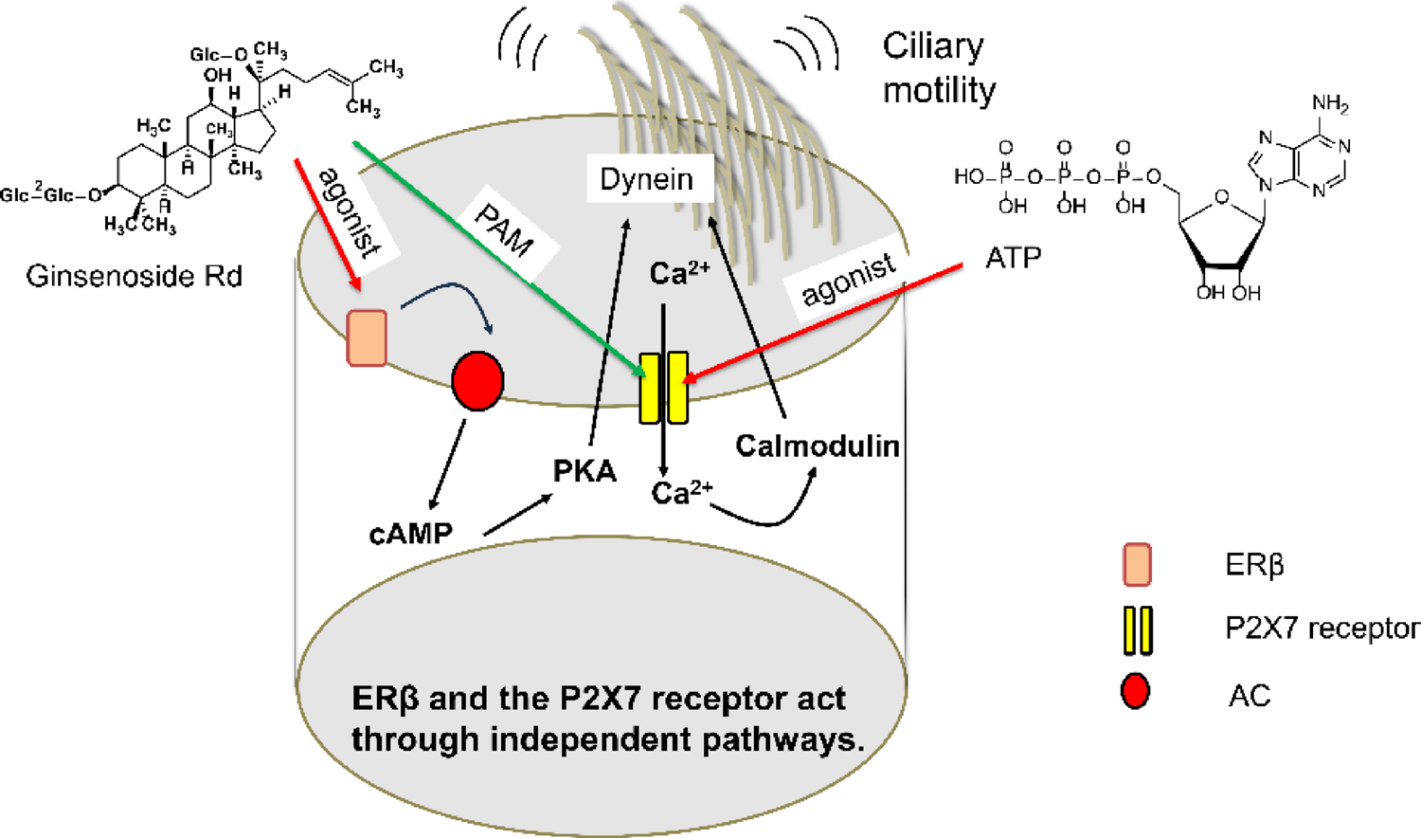
Dual Mechanism of Action of Ginsenoside Rd on Airway Ciliary Motility. Ginsenoside Rd (Rd) enhances ciliary motility through the activation of two independent signaling pathways. (1) Estrogen receptor (ER) β pathway: Rd acts as an agonist for the ERβ. This effect leads to an increase in intracellular cAMP levels and subsequent activation of PKA, thereby stimulating ciliary motility. (2) P2X7 receptor pathway: Rd acts as a positive allosteric modulator of the P2X7 receptor, independently of the ERβ pathway. In the presence of ATP, Rd potentiates ATP-induced Ca^2+^ influx through the P2X7 channel. As a result, elevation of intracellular Ca^2+^ level activates the Ca^2+^-dependent pathway, thereby further enhancing ciliary motility. The β-D-glucopyranosyl group in the Rd structure was designated as glc

In previous reports, the effects of several ginsenosides and their derivatives on [cAMP]_i_ were investigated using A549 PinkFlamindo cells, which were established by introducing the PinkFlamindo cAMP-dependent fluorescent gene into A549 cells, a cell line also used as an airway epithelial model [69, 70]. Rd exhibited a particularly significant increase in [cAMP]_i_ compared to other ginsenosides and their derivatives, and also showed an increase in CBF [70]. Therefore, this section focuses on Rd. Rd enhances CBF in a concentration-dependent manner by acting as an ERβ agonist. This effect involves activation of PKA via elevation of [cAMP]_i_ and occurs within 20 min, suggesting a non-genomic effect [70]. Because E2 similarly increases CBF via membrane ERβ, Rd is presumed to act through the same pathway [37]. Rd alone does not affect [Ca^2+^]_i_. However, in the presence of ATP, Rd also acts as a positive allosteric modulator (PAM) of the P2X7 receptor. Rd potentiates ATP-induced CBF-increasing effect following Ca^2+^ influx via P2X7 in a concentration-dependent manner [70]. Rd itself is not an agonist but a PAM of the P2X7 receptor. Moreover, this PAM’s effects were considered to occur independently of ERβ (Fig. 4). Recent studies revealed that PPD-type ginsenosides, including Rd, possess PAM activity at the P2X7 receptor, whereas PPT-type ginsenosides do not [71]. Additionally, the enhancement of the ATP response by Rd is significantly reduced by mutations in S59A, S60A, D318L, and L320A of the P2X7 receptor [72]. These may be allosteric modulator binding sites for Rd. The PAM action of Rd was also strictly regulated by modification of the triterpene skeleton. Importantly, no modification at C-6 is necessary [73]. It was also considered necessary to have a substituted group with a hydroxyl group, such as a sugar chain, at C-3 or C-20 [73]. PPD-type ginsenosides include ginsenoside Rb1, Rb2, Rb3, Rc, and Rd. Among these five ginsenosides, Rd has been reported to most effectively increase cAMP levels [70]. Since Rd exerts both ERβ agonist activity and PAM effects on P2X7 receptors to regulate [cAMP]_i_ and [Ca^2+^]_i_, it may be a promising compound for therapeutic applications.

### Therapeutic potential of ginsenoside Rd in chemotherapy-associated airway dysfunction

Here, we discuss the translational potential of Rd in restoring ciliary function impaired by chemotherapy, highlighting its possible role in preventing respiratory infections in cancer patients. Recently, it has been reported that the risk of respiratory infection increases in cancer patients, with chemotherapy recognized as a risk factor [74]. For example, analysis of Japanese health insurance claims data demonstrated an elevated incidence of respiratory infections in patients treated with docetaxel [29]. In contrast, the incidence of urinary tract infections remained unchanged in patients treated with docetaxel. These findings suggest that chemotherapy may induce respiratory infections by damaging airway epithelial tissue rather than through systemic side effects caused by leukopenia. Molecular insights into the effects of anticancer drugs on airway epithelial tissue have been limited. However, recent studies have reported that several anticancer drugs, including docetaxel, paclitaxel, doxorubicin, pemetrexed, and cisplatin, impair ciliary motility [75]. Although taxane anticancer drugs were shown to decrease [Ca^2+^]_i_, the precise molecular mechanisms underlying the reduction in ciliary beat frequency (CBF) induced by each anticancer drug remain poorly understood. Taxane anticancer drugs are known to inhibit microtubule depolymerization, doxorubicin has been reported to suppress intraflagellar transport protein 88 [76], and cisplatin is associated with the generation of reactive oxygen species [77]. These effects are thought to contribute to CBF disturbance. In contrast, Rd has been shown to restore anticancer drug–induced reductions in CBF to baseline levels or even enhance CBF beyond baseline. This rescue effect is mediated by Rd-induced increases in [cAMP]_i_ [75]. Although these observations were obtained from short-term experiments, they highlight the potential translational use of Rd. In the future, the adjunctive use of Chinese herbal medicines containing Rd, such as Ninjin-yoei-to, or newly developed Rd-based drugs during chemotherapy may reduce the risk of respiratory infections as a clinical complication.

## Translational and mechanistic considerations

Although airway ciliary motility is regulated by multiple signaling pathways, including intracellular cAMP, Ca^2+^, NO, and Cl⁻, only a limited number of compounds have been mechanistically characterized as ciliary modulators. Among these, ginsenoside Rd is relatively well defined, acting through ERβ activation and PAM of the P2X7 receptor [70]. Nevertheless, important gaps remain in translating these mechanistic insights into clinically meaningful interventions for human respiratory diseases.

First, despite its relatively well-characterized molecular targets, the current understanding of Rd-induced ciliary activation is largely based on in vitro or ex vivo studies. Evidence from in vivo models remains scarce, limiting evaluation of its physiological relevance. Moreover, most studies focus on short-term outcomes, leaving the long-term safety and potential pro-inflammatory consequences of sustained P2X7 receptor activation largely unexplored.

Second, it remains unclear whether pharmacological enhancement of ciliary motility alone translates into improved mucociliary clearance or reduced susceptibility to respiratory infections in disease settings. Although Rd has been shown to ameliorate chemotherapy-induced ciliary dysfunction [75], evidence from chronic airway diseases, such as COPD conditions associated with impaired ciliary function and high infection risk, is limited [78]. In chronic airway diseases like COPD, airway remodeling and goblet cell hyperplasia significantly reduce the density of ciliated cells. This structural deficit suggests that enhancing CBF alone may be insufficient to restore effective MCC. Therefore, therapeutic strategies should ideally aim not only to activate CBF but also to restore the ciliated cell population. In recent years, it has been reported that treatment with IL-6 and laser irradiation promotes the differentiation of airway epithelium and increases the number of ciliated cells [79, 80].

Third, pharmacokinetic limitations pose a translational barrier for natural compounds. Many natural compounds struggle to achieve effective concentrations in the airway epithelium due to their low systemic bioavailability [81]. Therefore, it is necessary to establish effective delivery strategies for the airways. For example, establishing methods to increase local concentrations on the surface of the airway, such as through inhalation, could potentially overcome the issue of systemic bioavailability. It is known that the major PPD-type ginsenosides are absorbed after being metabolized into compound K [82]. While Rd has been reported to accumulate in the lungs, the pharmacokinetics of compound K were reported to accumulate relatively higher concentrations in the liver of rats and mice [83]. This suggests that the metabolites may exert different effects. Furthermore, there is no evidence whether compound K is a PAM of the P2X7 receptor, and its effect on increasing CBF may not be as promising as Rd. Based on this background, increasing the local concentration of Rd in the airways via inhalation may also be a promising approach.

Fourth, it is necessary to research mucus secretion volume and mucus viscosity. MCC not only involves ciliary movement but also involves a significant contribution of mucus. It is generally reported that an increase in CBF is proportional to mucus transport and that CBF contributes significantly to MCC [84, 85]. However, when mucus volume and viscosity are high, an increase in CBF alone is insufficient [84]. From this perspective, carbocysteine, which modifies viscosity, can be considered a useful therapeutic agent. Either way, it will become important to investigate the effects on mucus of compounds reported to increase CBF.

Collectively, these considerations highlight both the promise and the current limitations of pharmacological ciliary activation by natural compounds. Despite these limitations, natural compounds have historically made significant contributions to drug development. They remain a source of clinically useful therapeutic agents today and constitute a large proportion of FDA-approved pharmaceuticals [86, 87]. It is expected that the development of ciliary motility activators will progress through further technological and research advancements.

## Conclusion

This review summarizes current knowledge on the regulation of airway ciliary motility together with recent evidence on endogenous mediators, pharmaceutical agents, and natural compounds that enhance ciliary function. These insights support the potential of targeting ciliary motility, particularly through approaches derived from natural products and herbal medicines, as a meaningful and underexplored strategy to enhance MCC. Future studies employing disease models and in vivo systems will be required to clarify the physiological relevance and therapeutic potential of this emerging approach.

## Data Availability

No data was used for the research described in the article.

## Abbreviations

[Ca^2+^]_i_: Intracellular Ca^2+^ concentration
[cAMP]_i_: Intracellular cAMP concentration
[Cl^−^]_i_: Intracellular Cl^−^ concentration
ACh: Acetylcholine
CBA: Ciliary bend angle
CBD: Ciliary bend distance
CBF: Ciliary beat frequency
CFTR: Cystic fibrosis transmembrane conductance regulator
COPD: Chronic obstructive pulmonary disease
E2: 17β-estradiol
ER: Estrogen receptor
GPCR: G protein-coupled receptors
MCC: Mucociliary clearance
NBC: Na⁺-HCO₃⁻ cotransporter
NO: Nitric oxide
PAM: Positive allosteric modulator
PCD: Primary ciliary dyskinesia
PDE: Phosphodiesterase
pH_i_: Intracellular pH
PKA: Protein kinase A
PKC: Protein kinase C
PKG: Protein kinase G
PPD: Protopanaxadiol
PPT: Protopanaxatriol
Rd: Ginsenoside Rd
RGAE: Red ginseng aqueous extract
SAC: Sensitive soluble adenylyl cyclase
T2R: Taste family type 2 receptors
